# Supplementary material for: Ability of Carotid Corrected Flow Time to Predict Fluid Responsiveness in Patients Mechanically Ventilated Using Low Tidal Volume after Surgery
Source: J Clin Med. 2021 Jun 17;10(12):2676. doi: 10.3390/jcm10122676 (PMC8234831; doi:10.3390/jcm10122676)
Supplement: Supplementary file 1 [file jcm-10-02676-s001.zip › jcm-1225953-suppl.pdf]

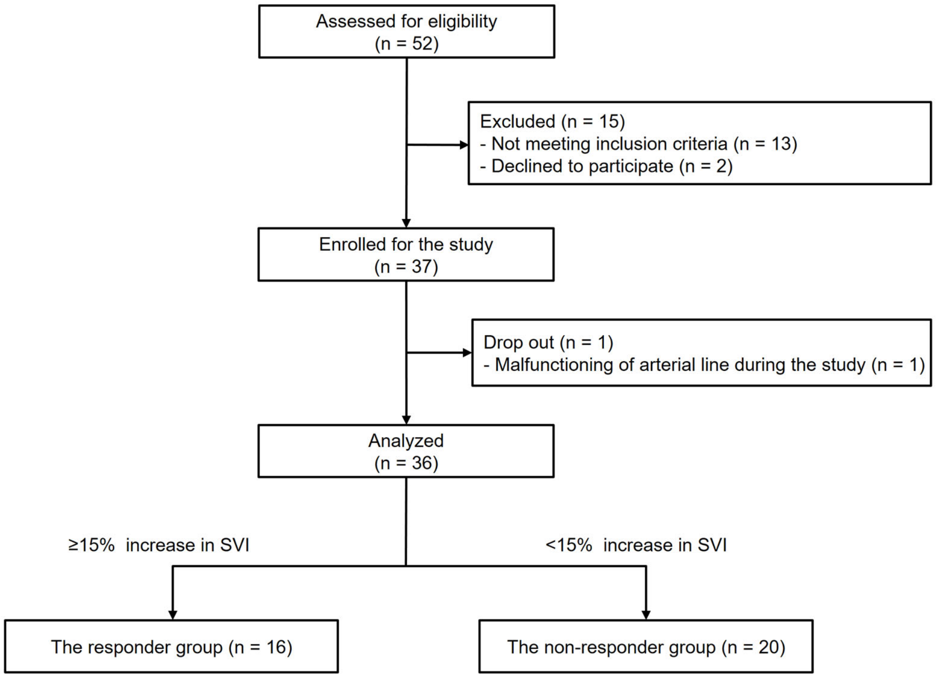

Figure S1. Flow chart of patient enrolment. SVI = stroke volume index.

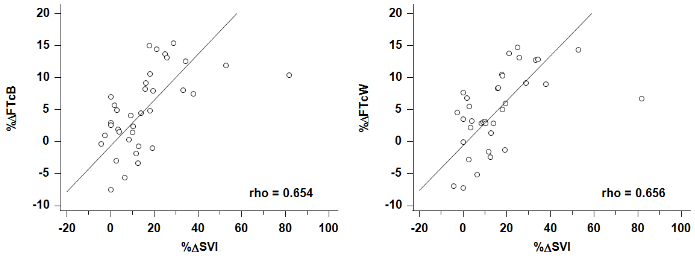

Figure S2. The relationship between the percent changes in stroke volume index and carotid corrected flow time from baseline to after fluid loading. Δ = change; FTcB = corrected flow time in the carotid artery calculated by Bazett's formula; FTcW = corrected flow time in the carotid artery calculated by Wodey's formula; SVI = stoke volume index.

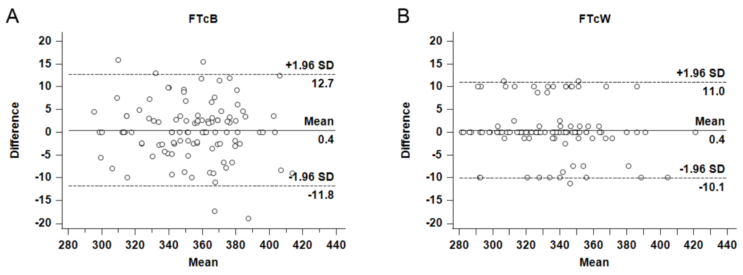

Figure S3. Bland-Altman analysis for the inter-observer agreement of the corrected flow time. The continuous lines indicate the mean difference (bias), and the dotted lines indicate the 95% limits of agreement (1.96 × SD). FTcB = corrected flow time in the carotid artery calculated by Bazett's formula; FTcW = corrected flow time in the carotid artery calculated by Wodey's formula.

Table S1. Clinical indicators suggesting hypovolemic status.

**Clinical indicator**
Systolic blood pressure of <90 mmHg
Mean arterial pressure of <70 mmHg or a decreased of >20% as compared to the preoperative status
Heart rate of >100 beats per minute
Urine output decreased by <0.5 mL/kg over 1 h
Lactate level in the arterial blood of >2 mmol/L
Capillary refilling time of >3 s

At least one of the following clinical signs must have been satisfied by the attending physicians to initiate fluid resuscitation.
